# Perceived social support and longitudinal trajectories of depression and anxiety in World Trade Center responders

**DOI:** 10.1007/s00127-023-02569-y

**Published:** 2023-10-24

**Authors:** Lisa J. Pijnenburg, Tjasa Velikonja, Robert H. Pietrzak, Jonathan DePierro, Lieuwe de Haan, Andrew C. Todd, Christopher R. Dasaro, Adriana Feder, Eva Velthorst

**Affiliations:** 1 Department of Psychiatry, Academic Medical Center, University of Amsterdam, Amsterdam, The Netherlands; 2 GGZ Rivierduinen, Institute for Mental Health Care, Leiden, The Netherlands; 3 Department of Psychiatry, Icahn School of Medicine at Mount Sinai, New York, NY USA; 4 Essex Partnership University NHS Foundation Trust, Runwell, UK; 5 U.S. Department of Veterans Affairs National Center for Posttraumatic Stress Disorder, VA Connecticut Healthcare System, West Haven, CT USA; 6 Department of Psychiatry, Yale School of Medicine, New Haven, CT USA; 7 Department of Social and Behavioral Sciences, Yale School of Public Health, New Haven, CT USA; 8 Center for Stress, Resilience and Personal Growth, Icahn School of Medicine at Mount Sinai, New York City, NY USA; 9 World Trade Center Health Program General Responder Data Center, Department of Environmental Medicine and Public Health, Icahn School of Medicine at Mount Sinai, New York, NY USA; 10 GGZ Noord-Holland-Noord, Institute for Mental Health Care, Heerhugowaard, The Netherlands

**Keywords:** Depression, Anxiety, Resilience, Social support, Disaster, World Trade Center attacks

## Abstract

**Purpose:**

While severely distressing events are known to affect mental health adversely, some survivors develop only short-lived or no psychiatric symptoms in the aftermath of a disaster. In the WTC Health Program General Responder Cohort (WTCHP GRC) we examined whether social support was protective against the development of depression or anxiety symptoms after the 9/11 WTC attacks and explored in a subsample whether trait resilience moderated this relationship.

**Methods:**

We analyzed data from 14,033 traditional and 13,478 non-traditional responders who attended at least three periodic health monitoring visits between 2002 and 2019. Linear mixed-effects models were used to examine depression (Patient Health Questionnaire-9; PHQ-9) and anxiety (Generalized Anxiety Disorder screener; GAD-7) scores. In a subsample of 812 participants, we also assessed if the association between social support and symptoms was moderated by an individual’s trait resilience level (Connor-Davidson Resilience Scale, CD-RISC).

**Results:**

For both traditional and non-traditional responders, perceived social support around 9/11 was associated with lower levels of depressive (β = − 0.24, S.E. = 0.017, *z* = − 14.29, *p* < 0.001) and anxiety symptoms (β = − 0.17, S. E. = 0.016, *z* = − 10.48, *p* < 0.001). Trait resilience scores were higher in responders with at least one source of social support during the aftermath of 9/11 compared to those without (mean 71.56, SD 21.58 vs mean 76.64, SD 17.06; β = 5.08, S.E. = 0.36, *p* < 0.001). Trait resilience moderated the association between social support and depressive (*p* < 0.001) and anxiety trajectories (*p* < 0.001) for traditional responders.

**Conclusion:**

Our findings suggest that perceived social support around a severely distressing event may have long-term protective effects on symptoms of depression and anxiety.

**Supplementary Information:**

The online version contains supplementary material available at 10.1007/s00127-023-02569-y.

## Introduction

Epidemiological and clinical studies indicate that around 70% of the general population worldwide is exposed to at least one potentially traumatic lifetime event [1, 2]. A study including nearly 70,000 individuals across the world showed that traumatic events include war-related trauma (13.1%), natural disasters (7.4%) and man-made disasters (4.0%) [2]. Importantly, while severely distressing events are known to affect mental health adversely [3], some survivors develop only short-lived or no psychiatric symptoms in the aftermath of the event [2, 3].

Arguably, among the most significant of man-made disasters of modern times in the Western world are the September 11^th^ attacks on the World Trade Center (WTC). The attacks posed enormous challenges for tens of thousands of professional rescue workers (e.g., policemen) and civilians (e.g., construction workers) involved in rescue, recovery and clean-up efforts (“WTC responders”) [4–6]. While a substantial proportion of WTC responders developed lasting mental health symptoms, including post-traumatic stress symptoms [4, 5, 7–9], depression and anxiety [6, 8, 10], others did not report significant symptoms and maintained their pre-disaster level of functioning, during the first six months [4, 11] and up to at least six years to 12 years after the attacks [5, 6, 9].

Studies have demonstrated that a strong social support network protects against the development of mental health problems [11–17] by mitigating the impact of stress on an individual [18–22]. The beneficial effect of social support is not limited to mitigating the risk for PTSD, but it can also buffer against the development of other mental health symptoms or disorders [11, 17, 23–25]. For example, higher levels of social support were found to be associated with lower rates of depression and anxiety after a large earthquake in Wenchuan, China [26, 27], fewer symptoms of PTSD and depression after the flood in Kashmir in 2014 [28] and fewer symptoms of PTSD, depression and anxiety in a group of rehabilitation and reconstruction workers following the 2005 earthquake in Northern Pakistan [29].

Social support has also been widely shown to be associated with a greater level of trait resilience across trauma-exposed populations, which may partly explain why social contact has been consistently identified as one of the key factors associated with more favorable PTSD symptom trajectories after 9/11 in first-responders [5, 6, 30], as well as in the general population [31]. It has yet to be investigated whether trait resilience may be an important moderator in an association between social support and symptoms of depression and anxiety symptoms in first responders as well.

Connor and Davidson [32] describe trait resilience as a set of personal qualities (e.g., being able to adapt to change) that enables a person to “bounce back” after facing some form of adversity. Importantly, while the role of perceived social support in mitigating PTSD symptom development after the 9/11 attacks has been investigated [6], its role in the development and long-term trajectories of depression and/or anxiety symptoms after the 9/11 attacks has remained largely unaddressed.

The present study will be the first to examine whether a social support network may also be associated with more favorable depressive or anxiety trajectories after the 9/11 WTC attacks. WTC responders participated in the WTC Health Program General Responder Cohort (WTCHP GRC) where they completed assessments during periodic health monitoring visits for almost two decades, providing a unique opportunity to explore the potentially protective effects of a social support network on longitudinal trajectories of depressive symptoms and anxiety.

Our first aim was to examine the association between perceived social support and longitudinal trajectories of depressive symptoms and anxiety in WTC responders. In secondary analyses, we explored whether an association between social support and favorable longitudinal trajectories of depressive and anxiety symptoms was moderated by a higher level of trait resilience.

## Methods

### Participants

For the present study, we analyzed data from 27,511 members of the WTCHP GRC, who attended at least three periodic health monitoring visits between 2002 and 2019. The WTCHP is a regional, clinical consortium that provides medical and mental health monitoring and treatment for certified conditions to WTC responders. Funded by the Centers for Disease Control and Prevention (CDC) and the National Institute for Occupational Safety and Health (NIOSH), the WTCHP recruits participants via outreach efforts that include union meetings, mailings, media articles, and some 50,000 phone calls in multiple languages [5, 6].

Eligibility for the WTC-HP requires having worked or volunteered as part of the rescue, recovery, restoration or clean-up efforts in (1) Manhattan south of Canal Street, (2) the barge-loading piers in Manhattan, or (3) the Staten Island landfill, for *at least 24 h* between 11 and 30 September 2001, OR for *more than 80 h* between 11 September and 31 December 2001 [5, 6, 33]. New participants were still joining the WTCHP at the time of this study, resulting in a wide range of dates for the first health monitoring visit (2002–2019). Eighteen months after the first visit, participants were eligible to return for a second visit, with subsequent visits initially scheduled every 18 months, and in recent years as frequently as every 12 months [33]. During each visit, a wealth of information was collected on physical and mental health prior to and after 9/11, employment, functioning, personality characteristics and the social environment.

Participants included WTC rescue and recovery workers. Police responders were considered ‘traditional responders’, whereas other responders (e.g. construction workers, cleaners, volunteers) were considered ‘non-traditional’ responders. Most firefighters attended a different CDC/NIOSH health monitoring program at the New York City Fire Department (FDNY) and are thus part of a separate cohort. See also Pietrzak et al. [6, 33]) and Feder et al. [5]) for more detailed descriptions of the study sample.

### Assessments

#### Depressive and anxiety symptoms

*Depressive symptoms* were assessed via the Patient Health Questionnaire-9 (PHQ-9) [34, 35], a self-reported measure of depression that consists of nine items corresponding with the *Diagnostic and Statistical Manual of Mental Disorders, Fourth Edition* (DSM-IV) [36] criteria for major depressive disorder. In this questionnaire, respondents are asked to rate each item (for example ‘Little interest or pleasure in doing things’) on a scale from 0 to 3 based on how much a symptom has bothered them over the last two weeks (0 = not at all, 1 = several days, 2 = more than half the days, 3 = nearly every day). The sum-score of the PHQ-9 ranges from 0 to 27 and is validated for use as a measure of the severity of depressive symptoms in the general population [35]. PHQ-9 scores of 5, 10, 15, and 20 represented mild, moderate, moderately severe and severe depression, respectively [34]. Cronbach’s α was 0.88 for traditional responders and 0.92 for non-traditional responders in our study.

The Generalized Anxiety Disorder Screener (GAD-7) [37] was used to measure *anxiety symptoms*. This self-reported questionnaire has been validated as a screening measure for generalized anxiety disorder as well as for other anxiety disorders in the general population [37]. The GAD-7 items correspond to the most prominent diagnostic features of the DSM-IV criteria for generalized anxiety disorder (GAD) [36]. Respondents are asked how often during the last two weeks they have been bothered by each of the seven core symptoms of GAD (e.g., feeling anxious, having trouble relaxing). Response options are 0 = not at all, 1 = several days, 2 = more than half the days and 3 = nearly every day. The GAD-7 total score ranges from 0–21, with scores of ≥ 5, ≥ 10 and ≥ 15 representing mild, moderate and severe anxiety symptom levels [37]. Cronbach’s α on GAD-7 items was 0.71 and 0.69 for traditional and non-traditional responders, respectively.

*Lifetime history* of depression, anxiety disorder or PTSD was measured by asking the participant if they had ever been diagnosed by a healthcare professional with either of these disorders (and, if yes, during which year). Only diagnoses predating 2001 were considered a ‘prior diagnosis’.

#### Perceived social support

During their first health monitoring visit to the WTC-HP, participants were asked to list all important sources of support while working on the WTC rescue and recovery effort. Important sources of support in participants’ close and immediate social network included their spouse, partner, child(ren), parent(s), other family, friend(s), coworkers and boss. For study purposes, perceived social support was dichotomized into ‘none’ (26% of the participants) versus ‘at least one source of social support’ (74% of the participants).

#### Trait resilience

A subsample of the study population (n = 812, all recruited during the first wave (2002–2006)) completed the Connor-Davidson Resilience Scale (CD-RISC) [32] during the third health monitoring visit, a scale shown to be a valid measure of *trait resilience* in the general population [32]. In this self-reported scale, respondents are asked to indicate how much they agree with each of 25 statements as they apply to them over the last month, on a five-point Likert scale (0 = not true at all, 1 = rarely true, 2 = sometimes true, 3 = often true, and 4 = true nearly all of the time). Examples of statements include: ‘I am not easily discouraged by failure’ and ‘I can deal with whatever comes my way.’ The CD-RISC (continuous) total score ranges from 0 to 100, with higher scores reflecting greater perceived resilience [32]. The Cronbach’s α for this scale was 0.96 for traditional and 0.97 for non-traditional responders within this subsample.

#### WTC-related trauma exposure

To measure the level of exposure to potentially traumatic WTC-related events, we used the composite WTC Exposure Index, as described by Pietrzak et al. [6]. WTC-related exposures included in this index were (1) early arrival (i.e. beginning work for the WTC effort between 11 and 13 September 2001); (2) being caught in the dust cloud; (3) working primarily/adjacent to the collapse site, known as the ‘pit’ or ‘pile’ during September 2001; (4) working more than the median number of hours on the WTC effort; (5) exposure to human remains (i.e. any exposure to human remains between 11 September 2001 and 30 June 2002); (6) involvement in search and rescue efforts during September to October 2001; (7) slept on site; (8) traumatic death of a colleague, family member or friend on 9/11; (9) being treated for an injury or illness while working on the WTC recovery effort; and (10) knowing someone who suffered an injury on 9/11. The WTC Exposure Index is the number of listed exposures and ranges from 0 to 10.

### Statistical analysis

#### Sample characteristics

Similar to prior reports on the WTCHP cohort [5, 6], separate analyses were performed for traditional and non-traditional responders, since non-traditional responders had no prior training in disaster response in contrast to traditional responders. For the current study, we created three different ‘visit cohorts’, according to the year of the first health monitoring visit (2002–2006; 2007–2013; 2014–2019), to minimize any systemic differences between responders that entered the study shortly or longer after the WTC attacks. Group differences in sample characteristics were examined using χ^2^ and t-tests. Sum scores on the PHQ-9 and GAD-7 questionnaires were *z* transformed so that β values throughout represent standardized effect sizes (ES), with values 0.2, 0.5 and 0.8 indicating small, medium and large ESs, respectively [38, 39]. These *z* scores were used in all statistical analyses. (See Supplemental Table S1 for the correlation matrix.)

#### The association between perceived social support, depression or anxiety

Linear mixed-effect models were used to examine differences in (i.e. changes in the slope of) PHQ-9 and GAD-7 *z-*scores over time between individuals who did and did not endorse social support (“group”) while working on the WTC effort. Effects of interest were group (with or without social support) main effects and the interactions between group and responder type. A statistically significant group main effect would indicate a difference in levels of depressive or anxiety symptoms between individuals with or without social support. A statistically significant interaction effect would mean a different effect of social support on mental health for traditional and non-traditional responders. Visit cohort for first visit, age at 9/11/2001, sex, race/ethnicity, WTC Exposure Index, and diagnosis of depression, anxiety and/or PTSD prior to 9/11/2001 were entered as covariates in all models.

To explore whether the effect of social support would differ for different sources of social contact, we performed separate linear mixed effect models for work-related sources of social support (co-worker and/or boss) and private (‘close’) sources of social support (spouse, partner, children, parents, other family, friends) separately.

#### The role of trait resilience in the association between social support, depression and anxiety

Within the subsample of participants that completed the CD-RISC, we applied linear mixed models including a CD-RISC-social support interaction to assess to what extent the association between social support and depressive symptoms and anxiety was moderated by an individual’s trait resilience level. Due to responder type differences, separate models for traditional and non-traditional responders were employed. Analyses were adjusted for age at 9/11/2001, sex, race/ethnicity, WTC Exposure Index and diagnosis of depression, anxiety and/or PTSD prior to 9/11/2001.

All analyses were conducted using Stata/MP 14.2 [40]. An adjusted p-value threshold of 0.008 (0.05/6, for 2 dependent variables (depression, anxiety symptoms) × 3 statistical models (main effects, responder type interaction effects, and trait resilience interaction effects)) was used in all models to account for multiple comparisons.

## Results

A combined total of 27,511 responders (14,033 traditional responders and 13,478 non-traditional responders) with a minimum of three visits, and with available data on social support and at an assessment of anxiety or depressive symptoms for at least one of these visits was included in the analysis. The number of visits per participant in our study ranged from 3 to 14 (mean 6.61, SD 2.85). (See Table 1 for baseline characteristics by responder type.)

**Table 1:** Baseline characteristics of the sample by responder type

		Non-traditional responders (n = 13,478)		Traditional responders (n = 14,033)		Statistics
		N or mean	% or SD	N or mean	% or SD	
Age at visit 1 (years)		46.4	10.2	43.0	7.6	t = − 31.13, *p* < 0.001, β = 0.184
Visit cohort						
2002–2006		8,110	60.2%	5,624	40.1%	χ^2^ = 1.1e+03, *p* < 0.001
2007–2013		4,201	31.2%	6,729	48.0%	
2014–2019		1,167	8.6%	1,680	11.9%	
Number of visits		6.8	2.9	6.5	2.7	t = − 10.04, *p* < 0.001, β = − 0.060
Age on 9/11/2001 (years)		41.3	9.5	36.3	6.8	t = − 51.10, *p* < 0.001, β = − 0.294
Sex						
Female		1,797	13.3%	2,107	15.0%	χ^2^ = 15.97, *p* < 0.001
Male		11,681	86.7%	11,926	85.0%	
Education						
High School or less		5,500	42.8%	2,048	15.0%	χ^2^ = 2.5e + 03, *p* < 0.001
More than High School		7,347	57.2%	11,644	85.0%	
Marital status^a^						
Married or partnered		9,143	67.9%	10,666	76.0%	χ^2^ = 238.61, *p* < 0.001
Widowed, separated or divorced		2,786	20.7%	2,034	14.5%	
Single		1,542	11.4%	1,331	9.5%	
Race/ethnicity						
White, non-Hispanic		7,806	57.9%	8,566	61.0%	χ^2^ = 179.98, *p* < 0.001
Black, non-Hispanic		1,454	10.8%	1,424	10.2%	
Hispanic		3,192	23.7%	2,555	18.2%	
Other		1,026	7.6%	1,488	10.6%	
Income						
< Median income		8,579	63.7%	6,050	43.1%	χ^2^ = 1.2e+03, *p* < 0.001
≥ Median income		4,899	36.3%	7,983	56.9%	
WTC Exposure Index (range 0–10)		3.5	1.9	4.9	2.0	t = 61.21, *p* < 0.001, β = 0.346
Diagnosed with depression before 9/11		341	2.5%	70	0.5%	χ^2^ = 192.75, *p* < 0.001
Diagnosed with Generalized Anxiety Disorder before 9/11/2001		238	1.8%	66	0.5%	χ^2^ = 105.59, *p* < 0.001
Diagnosed with PTSD before 9/11^b^		668	6.2%	365	3.7%	χ^2^ = 69.02, *p* < 0.001
Sources of social support while working on the WTC effort						
None		4,449	33.0%	2,729	19.4%	χ^2^ = 655.76, *p* < 0.001
At least one source of support		9,029	67.0%	11,304	80.6%	
Resilience score (n = 812)		80.0	15.3	72.8	19.5	t = 23.37, *p* < 0.0001

^a^Missing: n = 9

^b^Missing: n = 6891

The percentage of female participants was higher in the group of traditional responders (15.0% vs. 13.3%). Traditional responders more often continued education after high school (85.0% vs. 57.2%) and were more frequently married (76.0% vs 67.9%) compared to non-traditional responders. In addition, traditional responders were more often of White, non-Hispanic ethnicity (61.0% vs. 57.9%) than non-traditional responders, and their reported yearly income was more frequently higher than average for the cohort in the year of the first health monitoring visit (56.9% vs. 36.3%) than non-traditional responders. Furthermore, the WTC-related exposure severity was higher among traditional responders (4.9 vs. 3.5) and traditional responders were less likely to report a history of a diagnosed depression (0.5% vs 2.5%) or anxiety diagnosis (0.5% vs. 1.8%) before 2001 than non-traditional responders. Unsurprisingly, given that reporting a mental illness could affect a police officer’s fitness for duty, non-traditional responders more often reported a history of PTSD than traditional responders (6.2% vs. 3.7%). Traditional responders more frequently indicated having had at least one source of social support or more while working on the WTC effort compared with non-traditional responders (80.6% vs. 67.0%).

### Social support is associated with lower depressive or anxiety symptom levels over time

Overall, traditional responders reported fewer symptoms of depression (β = 0.72, S.E. = 0.024, *z* = − 29.91, *p* < 0.001) and anxiety (β = − 0.58, S.E. = 0.023, *z* = − 25.13, *p* < 0.001) compared with non-traditional responders. For both traditional and non-traditional responders, reporting at least one source of social support during the period around 9/11 was associated over time with significantly lower levels of depressive (overall effect: β = − 0.24, S.E. = 0.017, *z* = − 14.29, *p* < 0.001) and anxiety symptoms (overall effect: β = − 0.17, S. E. = 0.016, *z* = − 10.48, *p* < 0.001) over time (see Table 2).

**Table 2:** Visit cohort, responder type, social support and responder-by-social support interaction effect and depressive and anxiety symptom levels

Measure	Visit cohort effect			Responder type effect			Social support effect			Responder- by- social support interaction	
	Visit cohort	β (95% CI)	*p*	Responder	β (95% CI)	*p*	Social support	β (95% CI)	*p*	β (95% CI)	*p*
Depressive symptoms											
PHQ-9	2002–2006	–	–	Non-traditional	–	–	No source	–	–		
	2007–2013	− 0.028 (− 0.05, 0.00)	0.022	Traditional	− 0.717 (− 0.76, − 0.67)	< 0.001*	≥ 1 source	− 0.243 (− 0.28, − 0.21)	< 0.001*	0.146 (0.09, 0.20)	< 0.001*
	2014–2019	0.011 (− 0.74, 0.76)	0.978								
Anxiety symptoms											
GAD-7	2002–2006	–	–	Non-Traditional	–	–	No source	–	–		
	2007–2013	− 0.007 (− 0.03, 0.02)	0.552	Traditional	− 0.579 (− 0.62, − 0.53)	< 0.001*	≥ 1 source	− 0.171 (− 0.20, − 0.14)	< 0.001*	0.101 (0.05, 0.15)	< 0.001*
	2014–2019	0.287 (− 0.48, 1.06)	0.466								

Adjusted for age on 9/11/2001, sex, race/ethnicity, WTC-exposure index, diagnoses of PTSD, and depression and/or anxiety disorder prior to 9/11/2001; PHQ-9: Patient Health Questionnaire-9; GAD-7: Generalized Anxiety Disorder Screener

*p < 0.008 is considered significant

**Fig. 1:**
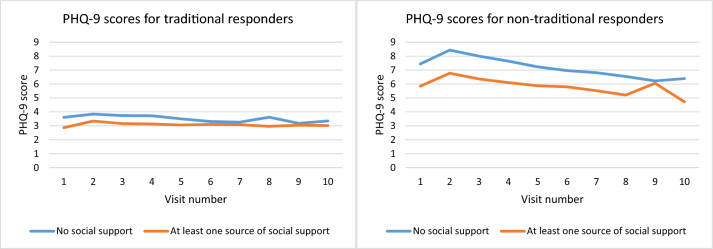
PHQ-9 scores for depressive symptoms and the availability of social support (none or one source of more) by type of responder

**Fig. 2:**
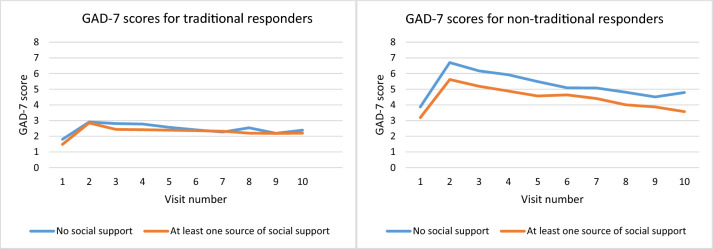
GAD-7 scores for anxiety symptoms and the availability of social support (none or one source of more) by type of responder

For both depressive and anxiety symptoms, we detected a significant interaction between responder type and perceived social support, suggesting that the association between perceived social support and depressive symptoms (β = 0.146, S.E. = 0.027, *z* = 5.48, *p* < 0.001) and between social support and anxiety (β = 0.101, S.E. = 0.026, *z* = 3.95, *p* < 0.001) was stronger in non-traditional compared with traditional responders (see Figs. 1 and 2).

Secondary analyses showed that results were comparable for work- related and close sources of social support (see supplemental figure S1 and S2 and table S3).

### Trait resilience as moderator in the association between social support and depressive and anxiety symptoms

A subsample of 812 individuals completed the (trait) resilience scale (CD-RISC) [32] at monitoring visit 3 (mean year 2007, SD 0.44). (See Supplemental Table S2 for baseline characteristics of this subsample.)

Trait resilience scores were higher in responders with at least one source of social support during the aftermath of 9/11 compared to those without (mean 71.56, SD 21.58 vs mean 76.64, SD 17.06; β = 5.08, S.E. = 0.36, *p* < 0.001). In regression models adding CD-RISC and social support as separate independent variables, both factors had an independent effect on symptom scores in traditional and non-traditional responders (see Table 3) with lower levels of trait resilience and lack of social support being associated with higher symptom levels.

**Table 3:** Association between trait resilience, social support and depression and anxiety symptoms

	Effect of trait resilience		Effect of social support		Trait resilience-by-social support interaction	
	β (95%CI)	*p*	β (95%CI)	*p*	β (95%CI)	*p*
Traditional responder						
Depression (PHQ-9)	− 0.014 (− 0.018, − 0.010)	< 0.001*	1.02 (0.645, 1.395)	< 0.001	− 0.011 (− 0.015, − 0.006)	< 0.001*
Anxiety (GAD-7)	− 0.013 (− 0.018, − 0.009)	< 0.001*	0.890 (0.490, 1.289)	< 0.001	− 0.009 (− 0.014, − 0.004)	< 0.001*
Non-traditional responder						
Depression (PHQ-9)	− 0.019 (− 0.021, − 0.016)	< 0.001*	0.128 (− 0.125, − 0.381)	< 0.001	− 0.004 (− 0.008, − 0.009)	0.014
Anxiety (GAD-7)	− 0.018 (− 0.020, − 0.017)	< 0.001*	− 0.117 (− 0.189, − 0.044)	0.002	− 0.002 (− 0.005, 0.002)	0.283

Adjusted for age at 9/11, type of responder, sex, WTC exposure index and previous diagnosis of anxiety or depression

*p < 0.008 indicates significance

Interestingly, for traditional responders there was an interaction effect between social support and trait resilience in the prediction of symptom levels (see Table 3), suggesting that the protecting effect of social support on symptom severity is stronger for responders with higher levels of trait resilience. No significant interaction effect was apparent for non-traditional responders.

## Discussion

In this large cohort of traditional and non-traditional 9/11 responders longitudinally followed for up to 20 years after the WTC attacks, we found that the availability of at least one important source of social support during participation in the WTC recovery effort was associated with fewer depression and anxiety symptoms in the years following the attacks; Interestingly, the association was most prominent for non-traditional responders. Police officers, although more heavily exposed to potential traumatic events during and in the aftermath of the disaster, reported fewer symptoms of depression and anxiety. However, even within this group, social support was associated with more favorable symptom trajectories over time. Notably, associations between social support and symptom severity was comparable for close and work-related contacts. Results from analyses in a subsample with available resilience scores suggest that trait resilience may moderate the association between social support and symptom severity in the non-traditional responder group.

Results of this study support earlier work from our group and others on the association between social support and psychiatric trajectories in traditional and non-traditional responders after the WTC attacks [5, 6, 9] and victims of other disasters [26, 27].

Overall, our findings add to the literature in several important ways:

First, building on work described in a review by Gariépy [12], which showed negative associations between social support and depression in the general population, results of our study suggest that the potential protective effect of perceived social support in rescue and recovery workers is not only long lasting, but it applies to both depressive and anxiety symptoms. Our findings are also largely in line with Adams et al. who conducted a study in WTC tower survivors. In their study, an association was found between low social support and long-term depression in WTC tower survivors, however, in their study low social support was not associated with PTSD and comorbid depression [41]. Additionally, the results of our study show that low social support is also associated with anxiety symptoms. These findings combined, suggest that social support mostly has a positive effect on ‘buffering’ chronic stress and less on acute stress during a traumatic event.

The most prominent theory explaining the positive effect of social support on the development of mental health symptoms is the ‘buffering hypothesis’ [18]. Social support is believed to mitigate the effects of stress on a person, thereby lowering the chance of developing mental health symptoms, such as depression or anxiety [18]. Our data supports the buffering hypothesis, since social support at the time of a disaster was associated with long-lasting positive symptom trajectories.

Notably, it is plausible that the effect of social support on mental health is bi-directional. While we adjusted for depression and anxiety diagnoses prior to 9/11, we cannot rule out any pre-existing mental health vulnerabilities. It may be that responders with robust mental health were able to make and maintain more social connections after the attacks, and were less likely to be affected by the traumatic events. Social contacts, in turn, may have prevented symptoms to develop in the years following.

Second, our study shows a positive association between social support and level of trait resilience, particularly in the non-traditional group that was vulnerable to develop depression and anxiety symptoms after 9/11. While social support and resilience were measured simultaneously in our study, and firm conclusions regarding directionality can therefore not be drawn, it is possible that social support may have enhanced the resilience of the non-traditional responder group. The idea that social support may mitigate the effect of stress after trauma through enhancing resilience is supported by a study among police officers after Hurricane Katrina, where resilience was also found to mediate the relationship between social support and symptoms of depression [42].

Third, our findings align with those of previous studies by our group and others, showing fewer PTSD symptoms in traditional 9/11 responders [5–7, 43], illustrating the differential impact that trauma may have on different parts of the labor force / population. Despite higher levels of WTC exposure among traditional responders, this group also reported fewer depression and anxiety symptoms over time compared with non-traditional responders.

There are several, non-exclusive factors that might contribute to this finding: self-selection of more resilient individuals in the police force [6, 7]; underreporting of feelings of depression or anxiety in traditional responders, similar to PTSD symptoms [7, 44]; fear of negative job consequences [9, 45–47]; or a higher level of preparedness in responding to major disasters [6, 48].

Last, our findings may have important clinical implications, particularly relating to social interventions in the aftermath of a disaster. The existing literature shows inconsistent effects of different interventions directed at increasing social support for the individual or within the broader community. Reviews focusing on interventions directed at increasing (perceived) social support in other stressful contexts show positive effects of social support interventions on the well-being of caregivers of people with dementia [49], post-stroke depression [50] and loneliness [51]. Hobfoll et al. [52] describe social connectedness as one of the five essential elements for immediate, post-disaster intervention, albeit with insufficient empirical literature to recommend specific intervention models. Our findings add to this body of work by suggesting that assistance in maintaining or creating social support from at least one person, for rescue and recovery workers exposed to the aftermath of mass trauma could also have great value in preventing long-lasting symptoms of depression and anxiety. Facilitating opportunities for social support in the aftermath of a traumatic event may be of particular value in high-risk work forces. Further research is needed to evaluate which interventions would be most suitable and effective for this population.

Some limitations on the findings from our study need to be considered. Since the WTCHP GRC is a medical monitoring and treatment program, data acquired is not specifically tailored to research and therefore limited in the level of details that is being questioned. We were able to examine only one self-reported indicator of presence or absence of perceived social support and researchers are encouraged to implement more detailed measures in future study designs. Another challenging feature of this study was the ongoing enrollment of study participants, the variation in number of health monitoring visits and the time intervals between these visits. By using ‘visit cohorts’ to cluster participants in three groups according to their first health monitoring visit, then examining the whole cohort over time, we tried to correct for the time factor as much as possible. Moreover, a recall bias may be present for participants who enrolled in the WTCHP during later years, as they might not remember their precise sources of social support around 9/11, or, in hindsight, they do not perceive social interactions as social support. This bias could be even more pronounced in those with higher levels of depressive symptoms or anxiety as these symptoms have been shown to both affect cognitive functions such as long-term memory [53], as well as someone’s social support network [54]. In contrast, the feeling of having been able to help and be appreciated by members of the community could have led to an increase in perceived social support during the time after 9/11 [55].

Lastly, we did not analyze the impact of additional factors over time after the attacks. It is unclear whether these factors resulted in accumulating trauma with long-lasting effects, or instead, made individuals more resilient against the effect of new-onset, significant life events [56].

Despite these limitations, results of our study emphasize the positive association between social support and depression and anxiety symptoms in first responders after a disaster. Our findings suggest that, to mitigate the negative long-term mental health effects stemming from exposure to mass trauma, it may be crucial to focus on the immediate social environment. Further research is needed to develop specific interventions aimed at enhancing perceived social support and screening for depression and anxiety symptoms in these populations, with different strategies of support developed for traditional and non-traditional responders.

### Supplementary Information

Below is the link to the electronic supplementary material.

Supplementary file1 (DOCX 59 KB)

## Data Availability

The data that support the findings of this study are available from the WTC Health Program General Responder Data Center (GRDC) at Mount Sinai. Restrictions apply to the availability of these data, which were used under a Data Transfer and Use Agreement for this study. Data are available via similar arrangements with the GRDC (Contact: christopher.dasaro@mssm.edu).
