# Supplementary material for: Perceived social support and longitudinal trajectories of depression and anxiety in World Trade Center responders
Source: Soc Psychiatry Psychiatr Epidemiol. 2023 Oct 24;59(8):1413–24. doi: 10.1007/s00127-023-02569-y (PMC11291574; doi:10.1007/s00127-023-02569-y)
Supplement: Supplementary file 1 — Supplementary file1 (DOCX 59 KB) [file 127_2023_2569_MOESM1_ESM.docx]

Supplement ‘Perceived social support and longitudinal trajectories of depression and anxiety in World Trade Center responders’

Authors:

Lisa J. Pijnenburg, MD^1,2^; Tjasa Velikonja, PhD^3,4^; Robert H. Pietrzak, PhD, MPH^5,6,7^; Jonathan DePierro, PhD^3,8^; prof. Lieuwe de Haan, PhD^1^; prof. Andrew C. Todd, PhD^9^; Christopher R. Dasaro, MA^9^; prof. Adriana Feder, PhD, MD^3^; Eva Velthorst, PhD^3,10^

Affiliations:

*^1^Department of Psychiatry, Academic Medical Center, University of Amsterdam, Amsterdam, the Netherlands*

*^2^GGZ Rivierduinen, Institute for Mental Health Care, Leiden, the Netherlands*

*^3^Department of Psychiatry, Icahn School of Medicine at Mount Sinai, New York, NY, United States of America*

*^4^Essex Partnership University NHS Foundation Trust, United Kingdom*

*^5^U.S. Department of Veterans Affairs National Center for Posttraumatic Stress Disorder, VA Connecticut Healthcare System, West Haven, CT, USA ^6^Department of Psychiatry, Yale School of Medicine, New Haven, CT, USA*

*^7^Department of Social and Behavioral Sciences, Yale School of Public Health, New Haven, CT, USA ^8^ Center for Stress, Resilience and Personal Growth, Icahn School of Medicine at Mount Sinai, New York City, NY, United States of America*

*^9^ World Trade Center Health Program General Responder Data Center, Department of Environmental Medicine and Public Health, Icahn School of Medicine at Mount Sinai, New York, NY, United States of America*

*^10^GGZ Noord-Holland-Noord, Institute for Mental Health Care, Heerhugowaard, the Netherlands*

Corresponding author:

Lisa J. Pijnenburg, MD

l.j.pijnenburg@amsterdamumc.nl

*Supplemental Table S1: Correlation matrix*

|  | **zGAD** | **zPHQ** | **Age 911** | **Age v1** | **Responder** | **Education** | **Marital status** | **Ethnicity** | **WTC exposure index** | **Sex** | **Depression** | **Anxiety** | **Visit cohort** | **Social support** | **Income** | **CDRISC** | **PTSD** |
| --- | --- | --- | --- | --- | --- | --- | --- | --- | --- | --- | --- | --- | --- | --- | --- | --- | --- |
| **zGAD** | 1.00 |  |  |  |  |  |  |  |  |  |  |  |  |  |  |  |  |
| **zPHQ** | 0.83 | 1.00 |  |  |  |  |  |  |  |  |  |  |  |  |  |  |  |
| **Age 911** | 0.06 | 0.08 | 1.00 |  |  |  |  |  |  |  |  |  |  |  |  |  |  |
| **Age visit 1** | 0.05 | 0.05 | 0.90 | 1.00 |  |  |  |  |  |  |  |  |  |  |  |  |  |
| **Responder** | -0.23 | -0.28 | -0.29 | -0.18 | 1.00 |  |  |  |  |  |  |  |  |  |  |  |  |
| **Education** | -0.11 | -0.16 | -0.11 | -0.06 | 0.31 | 1.00 |  |  |  |  |  |  |  |  |  |  |  |
| **Marital status** | 0.08 | 0.12 | -0.01 | -0.02 | -0.08 | -0.02 | 1.00 |  |  |  |  |  |  |  |  |  |  |
| **Ethnicity** | 0.04 | 0.09 | -0.07 | -0.13 | -0.08 | -0.12 | 0.09 | 1.00 |  |  |  |  |  |  |  |  |  |
| **WTC exposure index** | -0.01 | -0.03 | -0.10 | -0.13 | 0.35 | 0.13 | -0.05 | -0.09 | 1.00 |  |  |  |  |  |  |  |  |
| **Sex** | -0.07 | -0.07 | 0.02 | 0.02 | -0.02 | -0.06 | -0.24 | -0.17 | 0.10 | 1.00 |  |  |  |  |  |  |  |
| **Depression** | 0.08 | 0.08 | 0.06 | 0.02 | -0.08 | -0.01 | 0.06 | -0.01 | -0.02 | -0.05 | 1.00 |  |  |  |  |  |  |
| **Anxiety** | 0.06 | 0.06 | 0.04 | 0.00 | -0.06 | -0.01 | 0.03 | -0.02 | -0.01 | -0.02 | 0.40 | 1.00 |  |  |  |  |  |
| **Visit cohort** | -0.03 | -0.05 | -0.07 | 0.34 | 0.18 | 0.11 | -0.03 | -0.14 | -0.09 | 0.00 | -0.07 | -0.06 | 1.00 |  |  |  |  |
| **Social Support** | -0.08 | -0.12 | -0.08 | -0.08 | 0.15 | 0.13 | -0.09 | -0.07 | 0.19 | 0.02 | -0.00 | 0.00 | -0.02 | 1.00 |  |  |  |
| **Income** | -0.09 | -0.10 | 0.06 | 0.10 | 0.14 | 0.10 | -0.08 | -0.16 | 0.10 | 0.10 | -0.02 | -0.01 | -0.09 | 0.02 | 1.00 |  |  |
| **CD-RISC** | -0.40 | -0.46 | 0.02 | 0.01 | 0.19 | 0.19 | -0.11 | 0.01 | 0.06 | 0.00 | -0.12 | -0.07 | . | 0.12 | 0.11 | 1.00 |  |
| **PTSD** | 0.18 | 0.19 | 0.05 | 0.06 | -0.06 | 0.02 | 0.06 | -0.03 | 0.09 | -0.04 | 0.09 | 0.07 | 0.03 | -0.03 | -0.01 | -0.17 | 1.00 |

| *Supplemental Table S2: Baseline characteristics of the resilience subsample by responder type* | | | | | | |
| --- | --- | --- | --- | --- | --- | --- |
|  | | Resilience subsample  (n = 812) | | Not included (n = 27,986) | | Statistics |
|  |  | N or mean | % or SD | N or mean | % or SD |  |
| Age at visit 1 (years) |  | 43.4 | 8.8 | 44.9 | 9.2 | t = -4.59  p <0.001  β= -0.03 |
|  |  |  |  |  |  |  |
| Number of visits |  | 9.6 | 3.0 | 6.5 | 2.8 | t = 31.04  p <0.001  β= 0.18 |
|  |  |  |  |  |  |  |
| Visit cohort | 2002-2006 | 812 | 100% | 13,131 | 47.3% | χ^2^ = 877.11  p < 0.001 |
|  | 2007-2013 | - |  | 11,433 | 41.2% |  |
|  | 2014-2019 | - |  | 3,200 | 11.5% |  |
|  |  |  |  |  |  |  |
| Age at 911 (years) |  | 41.7 | 8.8 | 38.7 | 8.6 | t = 9.61  p <0.001  β = 0.057 |
|  |  |  |  |  |  |  |
| Sex | Female | 90 | 11.1% | 3,970 | 14.3% | χ^2^ = 6.69 p = 0.010 |
|  | Male | 722 | 88.9% | 23,794 | 85.7% |  |
|  |  |  |  |  |  |  |
| Education | High School or less | 241 | 30.2% | 7,528 | 28.3% | χ ^2^ = 1.47 p = 0.225 |
|  | More than High School | 556 | 69.8% | 19,098 | 71.7% |  |
|  |  |  |  |  |  |  |
| Marital Status | Married or partnered | 594 | 73.2% | 19,993 | 72.0% | χ ^2^ = 0.53 p = 0.767 |
|  | Widowed, Separated or Divorced | 135 | 16.6% | 4,860 | 17.5% |  |
|  | Single | 83 | 10.2% | 2,901 | 10.5% |  |
|  |  |  |  |  |  |  |
| Race/ethnicity | White, non-Hispanic | 591 | 72.8% | 16,418 | 58.7% | χ ^2^ = 75.78  p < 0.001 |
|  | Black, non-Hispanic | 62 | 7.6% | 2,908 | 10.4% |  |
|  | Hispanic | 130 | 16.0% | 5,796 | 20.7% |  |
|  | Other | 29 | 3.6% | 2,864 | 10.2% |  |
|  |  |  |  |  |  |  |
| Responder type | Non-Traditional | 497 | 62.0% | 12,981 | 48.6% | χ ^2^ = 55.68  p < 0.001 |
|  | Traditional | 305 | 38.0% | 13,728 | 51.4% |  |
|  |  |  |  |  |  |  |
| Income | < Median income | 313 | 38.5% | 15,363 | 54.9% | χ ^2^ = 85.03  p < 0.001 |
|  | ≥ Median income | 499 | 61.5% | 12,623 | 45.1% |  |
|  |  |  |  |  |  |  |
|  |  |  |  |  |  |  |
| WTC exposure index |  | 4.4 | 2.1 | 4.1 | 2.1 | t = 4.93  p <0.001  β = 0.029 |
|  |  |  |  |  |  |  |
| Diagnosed with depression before 9/11 |  | 24 | 3.0% | 398 | 1.4% | χ ^2^ = 12.85  p < 0.001 |
| Diagnosed with Generalized Anxiety Disorder before 9/11 |  | 22 | 2.7% | 288 | 1.0% | χ ^2^ = 20.92  p <0.001 |
| Diagnosed with Post-Traumatic Stress Disorder before 9/11 |  | 47 | 5.9% | 1,015 | 5.0% | χ^2^ = 1.12  p = 0.289 |
|  |  |  |  |  |  |  |
| Sources of social support around 9/11 | none | 178 | 21.9% | 7,621 | 27.2% | χ^2^ = 11.27  p = 0.001 |
|  | At least one source of support | 634 | 78.1% | 20,365 | 72.8% |  |
|  |  |  |  |  |  |  |
| PHQ-9 |  | 4.8 | 5.0 | 5.0 | 5.4 | t = -1.03  p = 0.302  β = -0.006 |
| GAD-7 |  | 3.6 | 4.4 | 3.6 | 4.6 | t = -0.12  p = 0.907  β = 0.000 |
|  |  |  |  |  |  |  |
| Resilience score  CD-RISC |  | 80.0 | 15.3 | 72.8 | 19.5 | t=23.37, p<.0001 |
|  |  |  |  |  |  |  |

*Supplemental table S3: form of social support and depression and anxiety symptom levels*

| Measure |  | Type of social contact | | Responder -by –close-support interaction | | Responder-by-work-support interaction | |
| --- | --- | --- | --- | --- | --- | --- | --- |
|  | Social support | *β (95%CI)* | *p* | *β (95%CI)* | *p* | *β (95%CI)* | *p* |
| Depressive symptoms |  |  |  |  |  |  |  |
| PHQ-9 | Close | -0.145 (-0.17, -0.12) | < 0.001* | 0.146 (0.09, 0.20) | < 0.001* | 0.178 (0.13, 0.22) | < 0.001* |
|  | Work | -0.126 (-0.15, -0.10) | < 0.001* |  |  |  |  |
|  |  |  |  |  |  |  |  |
| Anxiety symptoms |  |  |  |  |  |  |  |
| GAD-7 | Close | -0.098 (-0.12, -0.07) | < 0.001* | 0.102 (0.05, 0.15) | < 0.001* | 0.124 (0.08, 0.17) | < 0.001* |
|  | Work | -0.114 (-0.14, -0.09) | < 0.001* |  |  |  |  |
|  |  |  |  |  |  |  |  |
|  |  |  |  |  |  |  |  |
| *Close: social support from spouse, partner, child(ren), parent(s), other family and/or friend(s)*  *Work: social support from coworker and/or boss*  *Adjusted for age on 9/11/2001, sex, race/ethnicity, visit cohort, WTC-exposure index, diagnoses of PTSD, and depression and/or anxiety disorder prior to 9/11/2001 PHQ-9: Patient Health Questionnaire-9 GAD-7 : Generalized Anxiety Disorder Screener*  **p < 0.008 is considered significant* | | | | | | | |

*Supplemental figure S1: PHQ-9 scores and close versus work contacts among traditional and non-traditional responders*

*Supplemental figure S2: GAD-7 scores and close versus work contacts among traditional and non-traditional responders*
